# Immunity certification for COVID-19: ethical considerations

**DOI:** 10.2471/BLT.20.280701

**Published:** 2020-12-01

**Authors:** Teck Chuan Voo, Andreas A Reis, Beatriz Thomé, Calvin WL Ho, Clarence C Tam, Cassandra Kelly-Cirino, Ezekiel Emanuel, Juan P Beca, Katherine Littler, Maxwell J Smith, Michael Parker, Nancy Kass, Nina Gobat, Ruipeng Lei, Ross Upshur, Samia Hurst, Sody Munsaka

**Affiliations:** a Centre for Biomedical Ethics, National University of Singapore, Blk MD 11, 10 Medical Drive #02-03, Singapore117597, Singapore.; b Health Ethics and Governance Unit, World Health Organization, Geneva, Switzerland.; c Preventive Medicine Department, Federal University of São Paulo, São Paulo, Brazil; d Faculty of Law, University of Hong Kong, Hong Kong Special Administrative Region, China.; e Saw Swee Hock School of Public Health, National University of Singapore, Singapore, Singapore.; f Foundation for Innovative New Diagnostics, Geneva, Switzerland.; g Department of Medical Ethics and Health Policy, University of Pennsylvania, Philadelphia, United States of America.; h Centro de Bioética, Universidad del Desarrollo, Santiago, Chile.; i Faculty of Health Sciences, Western University, Ontario, Canada.; j Wellcome Centre of Ethics and Humanities, University of Oxford, Oxford, England.; k Berman Institute of Bioethics and Bloomberg School of Public Health, Johns Hopkins University, Baltimore, USA.; l Nuffield Department of Primary Care Health Sciences, University of Oxford, Oxford, England.; m School of Philosophy and Center for Bioethics, Huazhong University of Science and Technology, Wuhan, China.; n Dalla Lana School of Public Health, University of Toronto, Toronto, Canada.; o Institute for Ethics, History, and the Humanities, University of Geneva, Geneva, Switzerland.; p School of Health Sciences, University of Zambia, Lusaka, Zambia.

## Abstract

Restrictive measures imposed because of the coronavirus disease 2019 (COVID-19) pandemic have resulted in severe social, economic and health effects. Some countries have considered the use of immunity certification as a strategy to relax these measures for people who have recovered from the infection by issuing these individuals a document, commonly called an immunity passport. This document certifies them as having protective immunity against severe acute respiratory syndrome coronavirus-2 (SARS-CoV-2), the virus that causes COVID-19. The World Health Organization has advised against the implementation of immunity certification at present because of uncertainty about whether long-term immunity truly exists for those who have recovered from COVID-19 and concerns over the reliability of the proposed serological test method for determining immunity. Immunity certification can only be considered if scientific thresholds for assuring immunity are met, whether based on antibodies or other criteria. However, even if immunity certification became well supported by science, it has many ethical issues in terms of different restrictions on individual liberties and its implementation process. We examine the main considerations for the ethical acceptability of immunity certification to exempt individuals from restrictive measures during the COVID-19 pandemic. As well as needing to meet robust scientific criteria, the ethical acceptability of immunity certification depends on its uses and policy objectives and the measures in place to reduce potential harms, and prevent disproportionate burdens on non-certified individuals and violation of individual liberties and rights.

## Résumé

Les restrictions imposées dans le cadre de la lutte contre la pandémie de maladie à coronavirus 2019 (COVID-19) ont eu de lourdes conséquences économiques, sociales et sanitaires. Certains pays ont envisagé la mise en place d'une stratégie visant à alléger ces restrictions pour les individus guéris en leur octroyant un document communément appelé «passeport d'immunité». Ce document atteste qu'ils ont développé une immunité protectrice contre le coronavirus 2 du syndrome respiratoire aigu sévère (SARS-CoV-2), le virus à l'origine de la COVID-19. L'Organisation mondiale de la Santé a déconseillé l'usage du certificat d'immunité pour l'instant, car l'incertitude demeure quant à l'existence réelle d'une immunité à long terme pour ceux qui se sont remis de la COVID-19. En outre, la fiabilité des tests sérologiques censés déterminer si l'individu est immunisé n'est pas avérée. Un tel certificat ne peut être instauré que si les seuils scientifiques en matière d'immunité sont respectés, qu'ils soient fondés sur les anticorps ou sur d'autres critères. Néanmoins, même si le certificat d'immunité est désormais bien accepté par la science, il s'accompagne de nombreuses questions d'ordre éthique en ce qui concerne la limitation des libertés individuelles et la mise en œuvre. Dans le présent document, nous examinons les principales considérations à prendre en compte pour garantir l'acceptabilité éthique du certificat d'immunité visant à lever les mesures de restriction pour certaines personnes durant la pandémie de COVID-19. Cette acceptabilité éthique dépend non seulement de son degré de conformité à des critères scientifiques stricts, mais aussi de son usage, des objectifs politiques ainsi que des mesures mises en place pour atténuer les préjudices potentiels et éviter d'imposer une charge disproportionnée sur les individus dépourvus de certificat, ou de bafouer les droits et libertés de tout un chacun.

## Resumen

Las medidas restrictivas impuestas a causa de la pandemia de la enfermedad coronavirus de 2019 (COVID-19) han tenido graves efectos sociales, económicos y sanitarios. Algunos países han considerado la posibilidad de utilizar la certificación de inmunidad como estrategia para flexibilizar dichas medidas para las personas que se han recuperado de la infección mediante la expedición a dichas personas de un documento, comúnmente denominado pasaporte de inmunidad. Este documento certifica que han desarrollado inmunidad protectora contra el coronavirus-2 del síndrome respiratorio agudo severo (SARS-CoV-2), el virus que causa la COVID-19. La Organización Mundial de la Salud ha desaconsejado la aplicación de la certificación de la inmunidad en la actualidad debido a la incertidumbre sobre si existe realmente una inmunidad a largo plazo para quienes se han recuperado de la COVID-19 y a las preocupaciones sobre la fiabilidad del método de prueba serológica propuesto para determinar la inmunidad. La certificación de la inmunidad solo puede considerarse si se cumplen los umbrales científicos para asegurar la inmunidad, ya sea que se basen en anticuerpos o en otros criterios. Sin embargo, incluso si la certificación de la inmunidad llegara a estar bien respaldada por la ciencia, tiene muchas cuestiones éticas en cuanto a las diferentes restricciones de las libertades individuales y su proceso de aplicación. Examinamos las principales consideraciones sobre la aceptabilidad ética de la certificación de la inmunidad para eximir a los individuos de las medidas restrictivas durante la pandemia de la COVID-19. Además de necesitar cumplir criterios científicos sólidos, la aceptabilidad ética de la certificación de inmunidad depende de sus usos y objetivos de política y de las medidas que se apliquen para reducir los posibles daños y evitar que se impongan cargas desproporcionadas a las personas que no cuenten con dicha certificación y se violen las libertades y derechos individuales.

## ملخص

الإجراءات التقييدية المفروضة نظراً لأن جائحة مرض فيروس كورونا 19 (كوفيد 19) قد أدت إلى آثار اجتماعية واقتصادية وصحية وخيمة. اعتبرت بعض الدول استخدام شهادة المناعة كاستراتيجية لتخفيف هذه الإجراءات للأشخاص الذين تعافوا من الإصابة، وذلك بإصدار مستند لهؤلاء الأفراد، يُطلق عليها بصورة شائعة مصطلح: جواز المناعة. يشهد هذا المستند على أنهم يتمتعون بمناعة وقائية ضد المتلازمة التنفسية الحادة الوخيمة لفيروس كورونا 2 (SARS-CoV-2)، الفيروس المسبب لكوفيد 19. وقد نصحت منظمة الصحة العالمية بعدم تطبيق شهادة المناعة في الوقت الحالي بسبب عدم اليقين بشأن ما إذا كانت المناعة طويلة المدى موجودة بالفعل لدى هؤلاء الذين تعافوا من كوفيد 19، فضلاً عن المخاوف بشأن موثوقية طريقة الاختبار المصلي المقترحة لتحديد المناعة. لا يمكن النظر لشهادة المناعة بعين الاعتبار إلا إذا تم تلبية الشروط العلمية لضمان المناعة، سواء بناءً على الأجسام المضادة أو معايير أخرى. ومع ذلك، حتى إذا أصبحت شهادة المناعة مدعومة جيدًا من جانب العلم، فإنها تواجه العديد من القضايا الأخلاقية فيما يتعلق بالقيود المختلفة على الحريات الفردية وعملية تنفيذها. نحن ندرس الاعتبارات الرئيسية للقبول الأخلاقي لشهادة المناعة لإعفاء الأفراد من الإجراءات التقييدية أثناء جائحة كوفيد 19. وفضلاً عن الحاجة إلى تلبية معايير علمية صارمة، فإن القبول الأخلاقي لشهادة المناعة يعتمد على استخداماتها وأهدافها السياسة، والتدابير المتبعة لتقليل الأضرار المحتملة، ومنع الأعباء غير المتناسبة على الأفراد غير الحاصلين على الشهادة، وانتهاك الحريات والحقوق الفردية.

## 摘要

因新型冠状病毒肺炎（新冠肺炎）大流行而强制实施的限制性措施已造成严重的社会、经济和健康影响。有些国家已考虑采用免疫认证策略来放松这些限制性措施，即，向感染后康复的人员发放一份通常被称为免疫通行证的证件。该证件证明此类人员对引起新冠肺炎的病毒——严重急性呼吸综合征冠状病毒-2 (SARS-CoV-2)——具有保护性免疫力。世界卫生组织建议目前不要实施免疫认证，因为尚不确定感染新冠肺炎后康复的人员是否切实存在长期免疫力，且对计划用于测定免疫力的血清学检测方法的可靠性尚存疑虑。仅当达到确保免疫的科学阈值（无论是基于抗体还是其他标准）时，才可以考虑采取免疫认证。然而，即使免疫认证得到了科学的充分支持，也仍存在许多伦理问题，包括对个人自由的不同限制及其实施过程。对于在新冠肺炎大流行期间准许某些人员无需遵守限制性措施的免疫认证，我们研究了这种认证在伦理上予以接受的主要考虑因素。免疫认证不仅需要满足严格的科学标准，而且在伦理上是否予以接受还取决于免疫认证的用法、实施对象以及是否采取措施以降低对未获得认证的人员造成的潜在伤害，防止对其产生过重的压力以及违反个人自由和权利方面的规定。

## Резюме

Ограничительные меры, введенные с началом пандемии коронавирусного заболевания 2019 года (COVID-19), привели к серьезным социальным и экономическим последствиям, а также неблагоприятно отразились на здоровье населения. Некоторые страны рассматривают возможность использования сертификации иммунитета в качестве стратегии по ослаблению таких мер для людей, перенесших заболевание, посредством выдачи этим лицам документа, именуемого «паспорт иммунитета». Данный документ подтверждает, что эти лица обладают защитным иммунитетом к тяжелому острому респираторному синдрому коронавируса-2 (SARS-CoV-2) — вирусу, вызывающему COVID-19. Всемирная организация здравоохранения не рекомендует внедрять сертификацию иммунитета в настоящее время по причине неуверенности в формировании длительного иммунитета у лиц, переболевших COVID-19, а также ввиду опасений в отношении надежности предлагаемого метода серологического тестирования для определения иммунитета. Сертификацию иммунитета можно рассматривать только при условии соблюдения научных пороговых значений для обеспечения иммунитета, основанных на антителах или других критериях. Однако, даже если сертификация иммунитета получит широкую поддержку со стороны науки, она имеет массу этических проблем с точки зрения различных ограничений индивидуальных свобод и процесса реализации этих ограничений. Авторы исследуют основные соображения этической приемлемости сертификации иммунитета для освобождения людей от ограничительных мер во время пандемии COVID-19. Этическая приемлемость сертификации иммунитета зависит не только от необходимости соответствовать строгим научным критериям, но и от ее использования, целей политики и принимаемых мер по снижению потенциального вреда и предотвращению непропорционального бремени для не имеющих сертификата лиц и нарушений личных свобод и прав.

## Introduction

Most countries have adopted restrictive public health measures to control the coronavirus disease 2019 (COVID-19) epidemic caused by severe acute respiratory syndrome coronavirus-2 (SARS-CoV-2). These measures include physical distancing, closure of schools and businesses, requirement of face coverings and travel restrictions. While effective, the measures have severe social, economic and health effects.^1^ In the absence of a vaccine or specific effective treatments, countries such as Chile,^2^ Germany,^3^ Italy,^4^ United Kingdom of Great Britain and Northern Ireland,^5^ United States of America,^6^ and the city of Madrid in Spain,^7^ have expressed interest in immunity certification to exempt individuals who have recovered from COVID-19 from restrictive measures; Estonia has developed software to support the digital implementation of such certification.^8^ Immunity certification would provide individuals who have recovered from COVID-19 with a document – so-called passport or certificate – certifying them as immune to COVID-19 (or at low risk of reinfection) based on a reliable test: serological testing for antibodies against SARS-CoV-2 is the most commonly proposed method.

Population seroprevalence studies from several countries suggest that only a minority of the population is likely to be infected during the first epidemic wave of the pandemic.^9^^,^^10^ Immunity certification would therefore exempt only a small fraction of a country’s population from restrictive measures if applied now or in the near future. In general, governments are trying to find ways to ease some restrictions while also minimizing public health risk. For most countries, this easing has meant lifting restrictive measures gradually and in phases based on certain criteria such as epidemiological data, health system capacity, capacity for surveillance testing, isolation of infected individuals and contact tracing to suppress spread.^11^^–^^13^ In doing so, governments recognize that they may need to reverse course and impose more restrictive measures again, if public health data show increasing spread of SARS-CoV-2.

If recovery from COVID-19 does confer some degree of immunity, decisions on when and how to lift restrictive measures could also be informed by serosurveys to estimate the proportion of a population with potential immunity to SARS-CoV-2. In contrast to this population-based application, immunity certification applies testing – which may be based on, but is not limited to, serological testing – to identify individuals with protective immunity and lifting of restrictions specifically for these individuals. An immunity certification programme could complement population- or community-based strategies to ease restrictive measures, to secure societal and individual freedoms and well-being.

Currently, however, the extent and duration of antibody-mediated immunity to protect against SARS-CoV-2 reinfection have not been scientifically established.^14^^,^^15^ There are also concerns about the suitability of available serological tests to measure individual immunity to SARS-CoV-2. As such, the World Health Organization (WHO) has advised against the use of immunity certificates at this time as they have the potential to increase the risk of continued transmission.^16^

Three conditions for the scientific acceptability of immunity certification must be met for it to be a reasonable policy approach. First, the extent of protection conferred by antibody-mediated and cell-mediated immune responses to SARS-CoV-2 must be well understood, with reliable indicators of protection in terms of antibody titre or other relevant immune correlates. Second, declining immunity is common to coronavirus infections, including severe acute respiratory syndrome (SARS) and Middle East respiratory syndrome coronavirus (MERS-CoV),^17^^,^^18^ and cases of rapid reinfection with SARS-CoV-2 have been reported.^19^ Therefore, the minimum length of immunity against SARS-CoV-2 must be established and its duration monitored over time so as to understand whether and when certificate holders need to reassess their immunity status and, possibly, renew their certificate. Third, tests that can identify individuals who are immune must be available, and they must be sufficiently accurate and reliable to ensure false-positive and false-negative test results are within acceptable levels. What counts as an acceptable level of error is an inherently ethical issue about how much risk society is willing to accept. We return to this issue later in the paper.

Even if these scientific conditions are met, immunity certification programmes still present important ethical questions.^20^^–^^26^ The ethical acceptability of immunity certification depends on its uses and policy objectives as well as measures intended to reduce potential harms and prevent disproportionate burdens on non-certified individuals and the violation of individual liberties and rights. Different certification programmes raise different ethical concerns depending on: which activities would be affected; whether certification would be deployed for specific groups or occupations, or apply more widely to a population; whether certification would be required, recommended or optional; and the implications of not being certified. A policy designed to stratify individuals according to immunological risk will likely result in different tiers of restrictions on individual liberties, and potentially “undermine freedom to socialize and to travel, violate expectations of privacy, and exacerbate enforcement practices that discriminate against vulnerable groups”.^27^ In view of these issues, we discuss key justifications and considerations for the ethical acceptability of immunity certification for the purpose of exempting individuals from restrictive measures in response to the COVID-19 pandemic.

## Health certifications

Immunity certification for SARS-CoV-2 differs from other health certifications that may be used to exempt individuals from restrictive measures. In the current context where neither a vaccine nor an effective treatment specific to COVID-19 is available, testing for individual SARS-CoV-2 immunity would differ, for example, from tuberculosis or hepatitis B virus screening because people with tuberculosis or hepatitis B virus infection and those without prior evidence of exposure to hepatitis B virus could benefit from treatment or vaccination, respectively, to assure patient and occupational safety. We stress here that from an ethical, and legal, perspective, testing negative for protective antibodies should not have the same implications as testing positive for infection: individuals should not be regarded as an infectious disease threat simply because they lack immunity against SARS-CoV-2. In this regard, lack of immunity certification for SARS-CoV-2 should not be a barrier to health-care employment.

If used for international travel, immunity certificates should not be considered the same as health certificates that certify a negative COVID-19 status based on diagnostic testing (such as a polymerase chain reaction test), which some countries (e.g. China^28^ and Indonesia^29^) have implemented to reduce the risk of imported cases after resuming some international travel following the onset of the pandemic. If an effective vaccine is developed and becomes widely available, countries could require some incoming travellers to have an international certificate of vaccination (much like for yellow fever), and in a manner that is consistent with the requirements of the International Health Regulations (IHR).^30^ Potentially, a vaccination certificate and an immunity certificate could function similarly as required documents for international travel, especially if antibody-mediated (or T-cell-mediated) immunity and vaccination provide a comparable degree of protection.

A discussion on the ethics of international vaccination certification for COVID-19 is outside the scope of this paper. However, an immunity certificate differs from a vaccination or a negative COVD-19 test certificate as applied to international travel or other activities, because only those with a history of exposure to SARS-CoV-2 could obtain an immunity certificate. This has behavioural risk implications, as we discuss in the section on implementation concerns.

## Ethical justification

One justification for an immunity certification programme is the key public health principle of least infringement.^31^ This principle states that among available options to achieve a public health goal, policy-makers should choose the option that least infringes individual liberties. If it could be determined that some individuals are neither at risk of COVID-19 nor contagious, then there would be no justification for restricting their liberty. If there is a reliable method to validate individual immunity, it could in principle be used to identify people for whom no such justification existed.

A second justification for an immunity certification programme is the potential benefits conferred by such a programme. Immunity certification could be used for different purposes to achieve various individual and collective benefits.^20^^,^^22^^,^^26^ Some benefits are gained by the individuals who would receive the certificate. These individuals would be allowed to resume their usual activities; they would also be provided with the psychological assurance that, at least for some period of time, they are no longer susceptible to reinfection. At the same time, there are benefits to public health and public well-being that are gained by others. For example, immune-certified essential workers whose work requires or is best performed by face-to-face interactions (e.g. health care, collection of swab samples for diagnosis and surveillance, and elementary and secondary education) could interact more closely with people without fear of reinfection and onward transmission.

Lastly, immunity certification could allow certified individuals to safely interact with others who are isolated or where important relationships have been interrupted. Being able to visit and provide companionship to isolated people is valuable. Physical distancing has interfered with many person-to-person interactions. Most of these interactions are not seen as central to the COVID-19 response but the ability to resume physical and personal engagement with others would also be valuable.

## Ethical concerns 

Although an immunity certification programme could be ethically justifiable, it also raises ethical concerns about risk stratification based on immunological status and resultant differential easing of restrictive measures on individuals and groups.

In achieving a legitimate goal, such as protection of others from infectious disease risks, it is not necessarily unfair to treat individuals differently because of differences in relevant characteristics;^32^ indeed, this situation already exists with many employers providing special protections to employees in higher-risk groups (e.g. health-care workers). Nevertheless, by stratifying members of society into different tiers of risk of infection and contagiousness, an immunity certification programme may result in unequal treatment of individuals that is based on ethically irrelevant considerations of ethnicity, religion, socioeconomic status or similar differential traits. For example, members of a particular group X may come to be associated with having an innate immunity to SARS-CoV-2 because of a higher proportion of immune-certified individuals within that group relative to others. This apparent innate immunity may in fact be due to non-biological factors such as differences in exposure and infection rates and access to health care and immunity certification. Members of other groups, particularly those who are living with systemic discrimination and marginalization, may be barred from, or face more barriers to, accessing particular areas or activities if they are not immune-certified whereas members of group X would not face such barriers even if they are not immune-certified.

Immunity certification should not be used to dictate which individuals or groups can access an area or activity during the pandemic (or after it, if a vaccine is not available) when other measures, such as face masks, physical distancing and hand hygiene, can be implemented to reduce risks to an acceptable level. Certainly, the ability to exercise fundamental rights, such as voting, holding a public office or accessing health and social care and education, should never be dependent on having an immunity certificate. What does distinguish individuals with an immunity certificate is the extent to which they are required to conform to mitigation measures when accessing an activity, opportunity or right. For example, immune-certified individuals could be allowed to travel to other countries without the need for prior testing (for active infection) or quarantine within the period of certification, while people without certificates could be required to test and quarantine. The application of immunity certification in this context should conform to the IHR. The IHR allows State Parties to implement additional measures as a precaution, which should be based on scientific principles, evidence of risk to human health and any specific WHO guidance and advice. In addition, these measures must consider travellers’ human rights and not subject them to unfair discrimination (e.g. racial profiling).^23^ Furthermore, measures must not restrict international traffic or intrude on people’s lives more than reasonably available alternatives which would provide an appropriate level of health protection.

Other than preserving individual rights and basic liberties, different treatment of people based on immunological risk needs to be proportionate to the risk to be ethically acceptable.^33^^,^^34^ That is, the different costs and burdens imposed on individuals should not exceed the likely public health benefits, and should not result in disproportionate burdens on either the immune-certified or the non-immune individual. For example, making immune-certified health-care workers handle most of the work of the front-line COVID-19 response is unfair and unnecessary, as non-immune health-care workers could safely carry out front-line work with appropriate personal protective equipment. 

Governments that use immunity certification to ease restrictive measures for certain people should ensure that non-immune individuals who are having to isolate or maintain physical distance do not face a disproportionate number of problems. Governments can help these individuals by providing or increasing social support, as some places have done through income replacement, opportunities for remote social connections and work that can supplement income during the pandemic.

Even if governments do not use an immunity certification programme, private organizations and companies may use immunity certification for their purposes.^21^^,^^24^ A key question is which of these purposes are reasonably left to the discretion of private entities and which deserve scrutiny and, possibly, policy interventions to prevent negative effects on people. For example, insurers may charge non-immune-certified individuals higher premiums for specific insurance schemes. Governments should seek to prevent immunity certification policies – public and private – from making disadvantages worse in terms of opportunities for health care, employment, housing and so forth for particular populations. Private entities, on the other hand, should ensure that their use of immunity certification is fair and consistent with governmental policies that aim to minimize the worsening of social disparities as a result of the pandemic.

## Implementation concerns

For an immunity certification scheme to be ethically acceptable, attention also must be given to its implementation in the following areas.

### Test error

No test is perfect. All tests give false-negative and false-positive results and the degree of tolerable error must be determined. A predictable proportion of people tested will be told they have immunity to SARS-CoV-2 when in fact they do not and a proportion will be told they are not immune when in fact they are. A relevant ethical question therefore is what an acceptable proportion of false-negative and false-positive tests should be. This issue is an ethical task for which a satisfactory answer has yet to be offered. Nonetheless, with immunity certification, it is clearly more harmful for both the health risks of the individual and the public health risks to have false-positive results where people are told they are immune when they remain susceptible. Thus, the choice of test should be one with less chance of a false-positive result than a false-negative one if compromises have to be made.

Being clear about who should bear the responsibility for setting the threshold of tolerable error that will guide the use of these thresholds is therefore important. Private entities are motivated by their own interest and should not be allowed to set this threshold as their interests may not align with that of citizens and public health. This responsibility should therefore lie with governments who are accountable for protecting the interests of their citizens when making decisions about risk.

### Incentives and counterfeits

Depending on the benefits that immunity certification would provide, its implementation could result in perverse incentives: some individuals may intentionally increase their exposure to SARS-CoV-2 to become infected and thereby receive an immunity certificate. This behaviour would increase the risk of community spread and potentially cause serious illness or death to other people. Deliberate self-infection may be reduced to some extent by effective public communication on the risk of such behaviours to self and others. However, there would still probably be individuals who are willing to risk self-infection. The level of unintended harm that this behaviour could cause is likely to vary between settings and is difficult to predict. The degree to which sought-after benefits are still available to those without a certificate may ultimately be the best deterrent to such an incentive.

The perceived benefits of immune certificates could also result in a black market for counterfeit certificates. To reduce this risk, certification should be based on testing by an authorized body, results should be processed and confirmed by licensed laboratories and certificates should be issued by an appropriate health authority. Test results should also be securely linked to biometric identifiers or a protected digital identity to minimize fraudulent certificates.

### Privacy and stigma

Immune-certified and non-certified individuals would need to be identified for an immunity certification programme to work and be socially useful. While there may be limits to maintaining personal immunity certification information as private and confidential, measures should be implemented to minimize confidentiality breaches and non-consensual identification to reduce privacy concerns and protect non-immune-certified individuals from any potential stigma and harm. To minimize data abuse, immunity certificates, if kept in a database, should be controlled by a trusted agency. Legal and regulatory mechanisms should be in place to limit data access to legitimate governmental authorities and third parties and to intended purposes. In addition, data collection, processing and retention should be kept to the minimum necessary to achieve public health and socioeconomic objectives.

Privacy protections that comply with the IHR will also be needed at the international level if immunity certificates are used to facilitate interstate travel and trade. Any health information on an identified or identifiable person that is collected or received by a State Party under the IHR from another State Party or from WHO must be kept confidential and processed anonymously as required by national law. Furthermore, all reasonable steps must be taken to ensure that inaccurate or incomplete data are deleted or corrected.^30^

### Prioritization and costs

If immunity certification relies on serological tests, then people who had previously tested positive for COVID-19 and those who had not would need to undergo serological testing to be certified. Finite capacity and resources for testing means that, at least initially, prioritizing access to testing and immunity certification will be a necessary measure. This prioritization raises the question of fairness in test allocation.^24^^–^^26^ An immunity certification programme could increase socioeconomic disparities if there is inequitable access to antibody testing and certification due to affordability or accessibility issues. An allocation system should be established to maximize collective benefits, minimize risk and avoid increasing socioeconomic disparities. To promote fair allocation, access to testing and certification should be based on a centralized assessment of societal need rather than ability to pay. Priority should be given to sectors in which workers are essential or needed more urgently, as well as sectors in which workers (or volunteers) have greater contact with populations at higher risk of severe COVID-19. Individuals who are socially and economically worst off because of the pandemic could also be given priority if restrictive measures have badly affected their livelihoods, and if immunity certification is a way to restore their circumstances. 

Given that the implementation of restrictive measures and reducing their impacts are State responsibilities, there is a strong argument that the cost of testing for immunity certificates should be borne to some extent by the State, either directly or through employer-based financing schemes.^26^ Essential industries have an interest in ensuring access to antibody testing if it enables them to protect their workforce. Arguably, employers of essential workers have a shared responsibility in covering the cost of testing for their employees, and State resources could be used to support the cost of certification for workers in the informal sector and the self-employed.

In low-resource settings, particularly when only a small proportion of a country’s population is likely to have developed enduring natural protective immunity, States may justifiably decide not to cover the costs of immunity certification so as to prioritize other health and social needs or other strategies to ease restrictive measures for their population as a whole.

## Conclusion

Immunity certification, even where available and reliable, should never be used as the main strategy for reducing the effects of the COVID-19 pandemic. Individuals can still lead their lives, to varying degrees, with current public health measures for safe movement and assembly. 

If the scientific and ethical considerations outlined above are met, immunity certification could be used as a component of a plan that decreases the number of people subject to highly restrictive measures and increases the number able to take on certain higher-risk activities such as caring for others or providing needed services. Appropriate interventions should be implemented to protect the interests of individuals who are not immune-certified and who remain under severe restrictive measures. These interventions should be evaluated for their effectiveness and impact, including unintended secondary negative effects. Such an approach to the implementation of an immunity certificate programme would reduce disparities and prevent resulting disadvantages to those without an immunity certificate from becoming permanent.
